# How AI Can Help in the Diagnostic Dilemma of Pulmonary Nodules

**DOI:** 10.3390/cancers14071840

**Published:** 2022-04-06

**Authors:** Dalia Fahmy, Heba Kandil, Adel Khelifi, Maha Yaghi, Mohammed Ghazal, Ahmed Sharafeldeen, Ali Mahmoud, Ayman El-Baz

**Affiliations:** 1 Diagnostic Radiology Department, Mansoura University Hospital, Mansoura 35516, Egypt; daliamonir2525@gmail.com; 2 Bioengineering Department, University of Louisville, Louisville, KY 40292, USA; hekand01@louisville.edu (H.K.); a.sharafeldeen@louisville.edu (A.S.); ahmahm01@louisville.edu (A.M.); 3 Information Technology Department, Faculty of Computers and Informatics, Mansoura University, Mansoura 35516, Egypt; 4 Computer Science and Information Technology Department, Abu Dhabi University, Abu Dhabi 59911, United Arab Emirates; adel.khelifi@adu.ac.ae; 5 Electrical, Computer, and Biomedical Engineering Department, Abu Dhabi University, Abu Dhabi 59911, United Arab Emirates; maha.yaghi@adu.ac.ae (M.Y.); mohammed.ghazal@adu.ac.ae (M.G.)

**Keywords:** pulmonary nodule, artificial intelligence, deep learning, neural networks

## Abstract

**Simple Summary:**

Pulmonary nodules are considered a sign of bronchogenic carcinoma, detecting them early will reduce their progression and can save lives. Lung cancer is the second most common type of cancer in both men and women. This manuscript discusses the current applications of artificial intelligence (AI) in lung segmentation as well as pulmonary nodule segmentation and classification using computed tomography (CT) scans, published in the last two decades, in addition to the limitations and future prospects in the field of AI.

**Abstract:**

Pulmonary nodules are the precursors of bronchogenic carcinoma, its early detection facilitates early treatment which save a lot of lives. Unfortunately, pulmonary nodule detection and classification are liable to subjective variations with high rate of missing small cancerous lesions which opens the way for implementation of artificial intelligence (AI) and computer aided diagnosis (CAD) systems. The field of deep learning and neural networks is expanding every day with new models designed to overcome diagnostic problems and provide more applicable and simply used models. We aim in this review to briefly discuss the current applications of AI in lung segmentation, pulmonary nodule detection and classification.

## 1. Introduction

**Figure 1:**
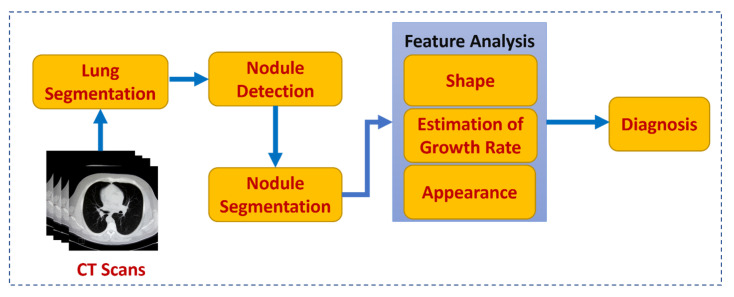
A Typical CAD System for Lung Cancer Diagnosis.

Lung cancer screening is a very important issue as the disease is the second most common type of cancer in both males and females. Lung cancer is responsible for 25% of all cancer cases in USA [1]. It is obvious that early detection was associated with a higher 5-year survival rate. Risk factors for developing lung cancer include all types of smoking (even electronic cigarettes and passive smoking) [2,3,4], family history either of single or multiple relatives especially those who developed cancer at young age [5], chronic obstructive lung disease [6], and human papilloma virus [7]. Recently, the United States Preventive Services Task Force recommended annual screening for lung cancer with low dose computed tomography (LDCT) for asymptomatic individuals aged 55 to 80 years who have a 30-pack year smoking history and currently smoke or have quit smoking within the past 15 years. Patients who have stopped smoking for 15 years, have a co-existing health problem limiting life expectancy, or are not candidates for surgical resection are excluded from annual screening. The algorithm of screening includes the number, the density, and size of solid, part solid or non-solid component of the nodules and according to these parameters, a follow-up schedule was designed [8,9]. Artificial intelligence was invented to enhance the computational abilities of computers and teach them to think, solve problems, and perform tasks in the same way as human beings. Recently, medical image analysis and diseases prediction and detection are among the most exciting applications of artificial intelligence. Using artificial intelligence techniques, computer aided diagnosis (CAD) systems have been developed and used in the analysis of medical imaging and have proved to be very helpful tools. AI techniques could be used to create a proper learning model to be used in clinical practice for lung cancer screening. The learning model should consist of four main steps; lung segmentation, followed by nodule segmentation/detection, then feature analysis, and the exclusion of false positive nodules (see Figure 1). Classification of detected pulmonary nodules into benign and malignant is based upon a preset of characteristic features including shape analysis, estimation of growth rate, and appearance analysis [10,11,12]. In this review, we will briefly discuss the current applications of AI in lung segmentation and pulmonary nodule detection and classification. This study reviews recent CT-based studies as well as studies published in the last two decades.

## 2. Lung Segmentation

**Figure 2:**
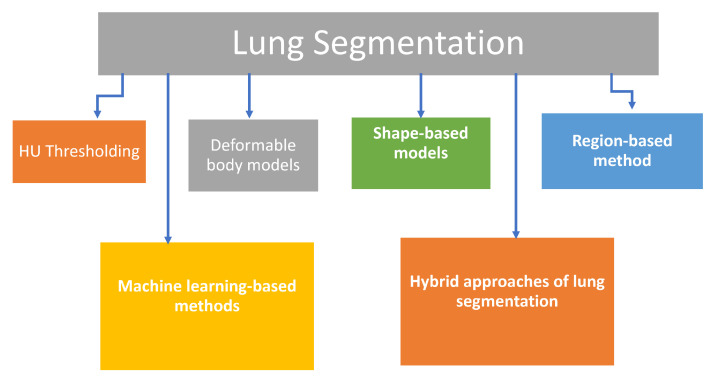
Main Categories of Lung Segmentation.

The first step in almost every CAD system dealing with lung disease is the segmentation. In this step, a preferred structure is delineated from its surrounding prior to analysis. Lung segmentation is very challenging due to different existing structures with near-similar densities such as the bronchi, bronchioles, pulmonary artery, and vein branches. Lung segmentation techniques can be categorized into four main categories based on: (1) Hounsfield unit (HU) threshold, (2) deformable boundaries, (3) shape models, (4) region/edge-based models, in addition to machine learning (ML) based methods and hybrid techniques which utilize a combination of methods to overcome the drawbacks of using single method (Figure 2). Details of the different categories are given below.

**Hounsfield unit (HU) thresholding:** Normal lung parenchyma displays low HU and appears hypodense in thoracic CT scan images in contrast to other structures such as heart, blood vessels or bronchial walls. Researchers tried to determine a threshold of HU to define lung parenchyma using different methods. Hu et al. [13] proposed a 3-step technique to perform lung segmentation. Their method started with extracting lung parenchyma utilizing a proper grey scale threshold. Then, separation of right and left lungs was performed using dynamic program. Lastly, a series of morphological operations were used to refine the pulmonary margins. This method was further used in the works of Ukil and Reinhardt [14], as well as Van Rikxoort [15]. Amato et al. [16,17] used grey scale thresholding once to extract the thorax from surrounding structures, and another time for extracting the lung from the rest of thoracic structures. A rolling ball algorithm is applied to lung periphery aiming not to miss any juxta-pleural nodule and exclude partial volume pixels. Pu et al. [18] designed an adaptive border marching (ABM) algorithm to reach the same purpose through refining lung margins. Gao et al. [19] proposed a 4-step method to separate the pulmonary vessels, and airways from lung parenchyma as well as separating right and left lungs based on a grey scale threshold. Other researchers used more sophisticated methods to define threshold used for lung extraction such as histogram analysis [20], and 3D fuzzy adaptive thresholding [21]. Limitations of lung segmentation using thresholding method are mainly related to its reliance on image resolution and type of scanners used (i.e., GE, Philips…). Another important issue is that there might be an overlap between densities of different lung structures making differentiation based on HU difficult.

**Deformable boundary models:** The second method used for lung segmentation is deformable boundary models including snakes, active contours, and level sets. These models start with an initial point then follow the shape of the desired structure influenced by internal and external forces. Itai et al. [22] utilized a 2D parametric deformable model to extract lung from computed tomography (CT) image using lung borders as an external guiding force. Silveria et al. [23,24] presented a technique that uses active contour and Level sets. They begin with a thresholding technique, then edge detection is initiated using a robust geometric active contour model around the lung. It divides into two and continues by multiple strokes which are categorized into valid and invalid according to confidence degrees. The major limitation of deformable boundary models is the high sensitivity of the selection of the initial point, in addition to inhomogeneity of lung structure that may lead to unsuccessful adaptation of lung boundaries [25].

**Shape-based models:** In this method, the stored data in the CAD system is used to improve the accuracy of lung segmentation. It utilizes either a statistical shape or lung appearance model. Unlike previously discussed methods, this approach of lung segmentation is more effective in dealing with lungs with moderate to severe pathology and with variations in lung anatomy as it gets benefit from trained models [26]. Sun et al. [27] proposed a 2-step lung segmentation technique that used a robust active shape model (RASM) matching method to segment the outline of the lungs guided by rib cage detection method, followed by using an optimal surface finding approach that was created by Li et al. [28] to fit the initial segmentation result to the lung. The right and left lungs were segmented separately. Sofka et al. [29] designed a multistage learning model that used predefined anatomical data to initiate a statistical shape model. Hau et al. [30] developed a graph-based search algorithm via cost function that takes into consideration the intensity, gradient, boundary smoothness, and rib anatomical information. Other researchers proposed a user interface framework [31] or Bayesian classification refined by Markov Gibbs Random Field (MGRF) method [32,33,34]. Similar approach was introduced by Chung et al. [35] who developed a Bayesian approach based on the Chan Vese (CV) model [36], where the data obtained from previous or upper frame image was used to predict lung image. False positive juxta-pleural nodule candidates were excluded via concave points detection and circle/ellipse Hough transform. Modification of lung contour by adding the final nodule candidates to the area of the CV model was the final step. More recently, Sun et al. [37] presented a new active shape model (ASM) algorithm to detect the outlier marker points by distance method aiming to get better assessment of lung periphery and juxta-pleural lung nodules. They also used a robust principal component analysis (RPCA) of low rank theory to remove noise from images in order to construct ASM. Despite the many advantages of shape model over other lung segmentation methods, its main limitation depends on the accurateness of the used stored data [25].

**Region-based method:** The main idea of region-based segmentation is that neighboring pixels in a certain region will have similar values [38]. An example of this method is the region growing method. If one pixel showed similar criteria to a predefined set then it is included in that region [38,39,40,41,42]. Other examples include watershed segmentation [43], random walks segmentation [44], graph cuts segmentation [45], and fuzzy connectedness [46]. This method of segmentation is suitable for homogenous structures such as lungs with no or mild pathology, airway and pathologic lesions with homogeneous density [25].

**Machine learning-based methods:** This method uses learning models composed of predefined measurable characteristics (called features) to identify normal and abnormal lung regions as well as different anatomical structures and finally construct the proper lung segmentation. Small image patches are labelled either as normal, abnormal, or neighboring soft tissue. The most common pathological patches used in clinical practice include consolidation, ground glass opacities, and fibrosis. A supervised training process uses data systems to extract features from each pixel/voxel and further classify them to predict lung field boundaries and reach final segmentation. A proper lung segmentation should include identification of both normal and pathological lung regions in the same process, and this is performed via examining each voxel in the CT image [47,48,49,50,51]. Multiple sophisticated algorithms were developed to reach this task, for example, Mansoor et al. [52] designed an ML algorithm that identifies a large spectrum of pulmonary pathologic lesions combined with region-based and neighboring anatomy guided correction segmentation. Obviously, this method is computationally expensive, but its remarkably high accuracy along with development of parallel computing and efficient well-processed workstations make this method feasible in clinical practice. One of the limitations of this method is that it uses small image patches which makes it impossible to predict structural information such as global shape of the lung. It is impossible to get feature data sets that can fit anatomical and physiologic lung variations in different subjects. Lastly, pixel by pixel assessment was the reason that this method had the least efficiency as compared to the other four major classes of lung segmentation [51,53,54,55,56].

**Hybrid approaches of lung segmentation:** No single lung segmentation method could fit with anatomical and pathological variants alone, this encouraged the development of combined approaches. As in the works of Mansoor et al. [52] and Hau et al. [30].

In summary, the literature reviews of lung segmentation system using these four different categories are presented in Table 1.

## 3. Pulmonary Nodule Detection and Segmentation

Lung cancer screening programs rely mainly on early detection of pulmonary nodules utilizing LDCT [71,72,73,74,75,76,77]. LDCT is capable of providing imaging of the thoracic region of high contrast, temporal, and spatial resolution in a very short acquisition time (single breath hold). However, detection of lung nodules is not as simple as it looks, as pulmonary nodules usually appear as a white spherical structure that could mimic a nearby small blood vessel or a collapsed bronchiole. In addition, the inter-reader variations in detection and the characterization of pulmonary nodules are merely subjective issues [10,78,79]. This opens the way for artificial intelligence and deep learning to overcome human errors and provide more effective procedures. The process of lung nodule detection passes into two stages; first detection of the pulmonary nodule candidates, second exclusion of the false positive nodules (FPN) and keeping only the true positive nodules (TPN). In other words, detection followed by classification [10,78,79].

**Computer-aided diagnosis (CAD) systems:** A large public database was generated to provide data that can be used to assess the performance of CAD detection and diagnostic systems and help further development. It is called the Lung Image Database Consortium and Image Database Resource Initiative (LIDC-IDRI). The creation of this database required great efforts as CAD was not used in annotation of images included [80]. Other databases such as data derived from the Dutch-Belgian NELSON lung cancer screening trial and LUNA16, LIDC, DSB2017, NLST, TianChi, and ELCAP datasets were utilized by most of the current research works dealing with CAD and deep learning (DL) [81]. The first step in the process of nodule detection is to unsharp the CT images by changing the image threshold which improves discrimination of pulmonary nodules from the rest of the surrounding lung parenchyma. A series of 3D cylindrical and spherical filters and template matching were used to detect small lung nodules [82,83,84,85,86,87,88,89]. However, the geometry of the candidate nodules doesn’t always fit into these spherical, cylindrical, or circular assumptions as it may be spiculated by its nature or due to attachment to nearby pleural surface or blood vessel [90]. Other studies proposed methods to detect lung nodules using k-means clustering technique [91,92,93] with further utilization of rule-based classifiers and linear discriminate analysis (LDA) to eliminate normal lung structures and reduce FPN. One study tried to solve the problem of eliminating an overlapping or contacting blood vessel by choosing a proper region of interest (ROI) in a 3-step model [94]. On the other hand, Oda et al. [95] and Siata et al. [96] used 3D algorithms; 3D filter by orientation map of gradient vectors and 3D distance transformation to overcome the same problem. Brown et al. [97] used prior patient images to create a specific model, so that any change in size and morphology of pulmonary nodules could be detected in follow up images easily. Messay et al. [98] used a fully automated CAD system that utilizes intensity thresholding and morphological operations to detect pulmonary nodules with a sensitivity of 82.66% with 3 FPN/scan. A set of 245 features was computed for each segmented lung nodule and Fisher Linear Discriminant (FLD) classifier was utilized. Similarly, Setio et al. [99] designed a CAD system to detect pulmonary nodules larger than 10 mm. They also used a multi-stage process of thresholding and morphological operations, then the extracted nodules were segmented and a set of 24 features was computed, finally the nodules were classified via a radial based vector supporting machine (VSM). A recent study aimed to solve the problem of using uncertain class data through the application of a CAD system based upon semi-supervised extreme learning machines (SS-ELM). This was done by using both certain class feature sets with labels, and unlabeled feature sets for training [100].

**Deep learning:** Deep learning is an advanced type of machine learning that uses complicated algorithms to model high level features and recognize characteristics. It is composed of statistical models that predict results depending on previous training on annotated or un-labelled datasets [101]. The algorithm could predict the presence of pulmonary nodule or predict its nature whether benign or malignant [102]. Convolutional neural network (CNN) is one of the most commonly used DL algorithms in the clinical practice. It was originally implemented in LeNet, which was designed by Yann LeCun et al. [103]. Since then, it gained more popularity and outperformed the existing state of the art texture analysis and support vector machine(SVM) methods. CNN model can build itself from the beginning even when dealing with new un-labelled features without the need for predefined set of features or complex human led pipes, in contrast to tissue radiomics or feature analysis. Another advantage of CNN over other methods is that all its components reach ultimate level at the same time, while in the case of tissue radiomics for instance, there is no guarantee that all components will fulfill high level. Additionally, it requires limited human supervision [10,104,105]. In the last decade, several research works emerged with different CNN algorithms and models designed for pulmonary nodule detection. Two studies showed exceptionally high accuracy (99–96.6%), sensitivity (97.5–96.9) and specificity (97.5–96.3). They proposed algorithms that either combined 2D and 3D artificial neural networks with intensity based statistical features [106] or used CAD system for different dimensions of angular histograms of surface normals (AHSN) features [107]. Other researchers used 2D and 3D subsets of features [108], local shape analysis and data-driven local contextual feature learning [109], geometric and intensity statistical features [110], or deep neural networks (DNN) [111]. Bergtholdt et al. [112] found that using support vector machine classifier improved the accuracy, sensitivity, and specificity of pulmonary nodule detection. One study [113] used deep believe network (DBN) to detect large nodules (>30 mm) with high accuracy of about 90%. Jakobs et al. [114] compared the performance of two commercial and one academic state of the art CAD systems and found that the updated commercial CAD system (Herakles) had the highest sensitivity reaching 82% with 3.1 FPN/scan. They found that about one third of the missed nodules were subsolid. They recommended the addition of a CAD scheme designed for subsolid nodules to improve the sensitivity of nodule detection. Another recent study reviewed several research works and found high sensitivity of DL algorithms when utilizing LUNA 16 dataset (in the range of 94.4–97%) with an average of 4 FPN/scan and LIDC-IDRI dataset (in the range of 80.06–94.1%) [115].

**Pulmonary nodule segmentation:** Nodule size is a strong predictor of neoplastic nature along with its progressive increase on follow up [116]. One large study demonstrated that risk of developing cancer in nodule less than 100 mm^3^ equals those with no nodules [117]. Nodule size was better assessed through volumetry rather than diameter as 2D measurements were found to be unreliable and showed wide inter and intra-observer variations [118]. Automated 3D measurement of pulmonary nodules provides better assessment of its morphology and growth rate [119]. Accurate nodule volumetry requires good nodule segmentation. Manual segmentation of lung nodules is time consuming and is far less accurate in comparison to deep learning semiautomated methods [120]. Most of the available algorithms concerned with pulmonary nodule detection rely on growing edge method where a predefined threshold acts as a seed that connects all nearby voxels of higher density [121]. As mentioned before, solid pulmonary nodules display higher density than surrounding lung parenchyma promoting easy discrimination by growing edge method, but difficulties occur when a vessel contacts or passes beside a pulmonary nodule or when it approximates the pleura [121,122]. The detection of ground glass nodules with indistinct margins is very problematic in manual segmentation. Tao et al., and Zhou et al., proposed novel methods via a multi-level statically based method [123] and a classifier by boosting k-nearest neighbor (kNN), whose distance measure is the Euclidean distance between the nonparametric density estimates of two regions [124]. Another more recent study segmented subsolid nodule through voxel classification that automatically eliminate blood vessels [125]. Other studies described more complex approaches to segment of pulmonary nodules of different densities and those with either vascular or pleural attachment via analysis of the core of the nodule [79,126,127]. Table 2 presents a summary of the state-of-the-art pulmonary nodule detection and segmentation systems.

## 4. Nodule Classification

**Figure 3:**
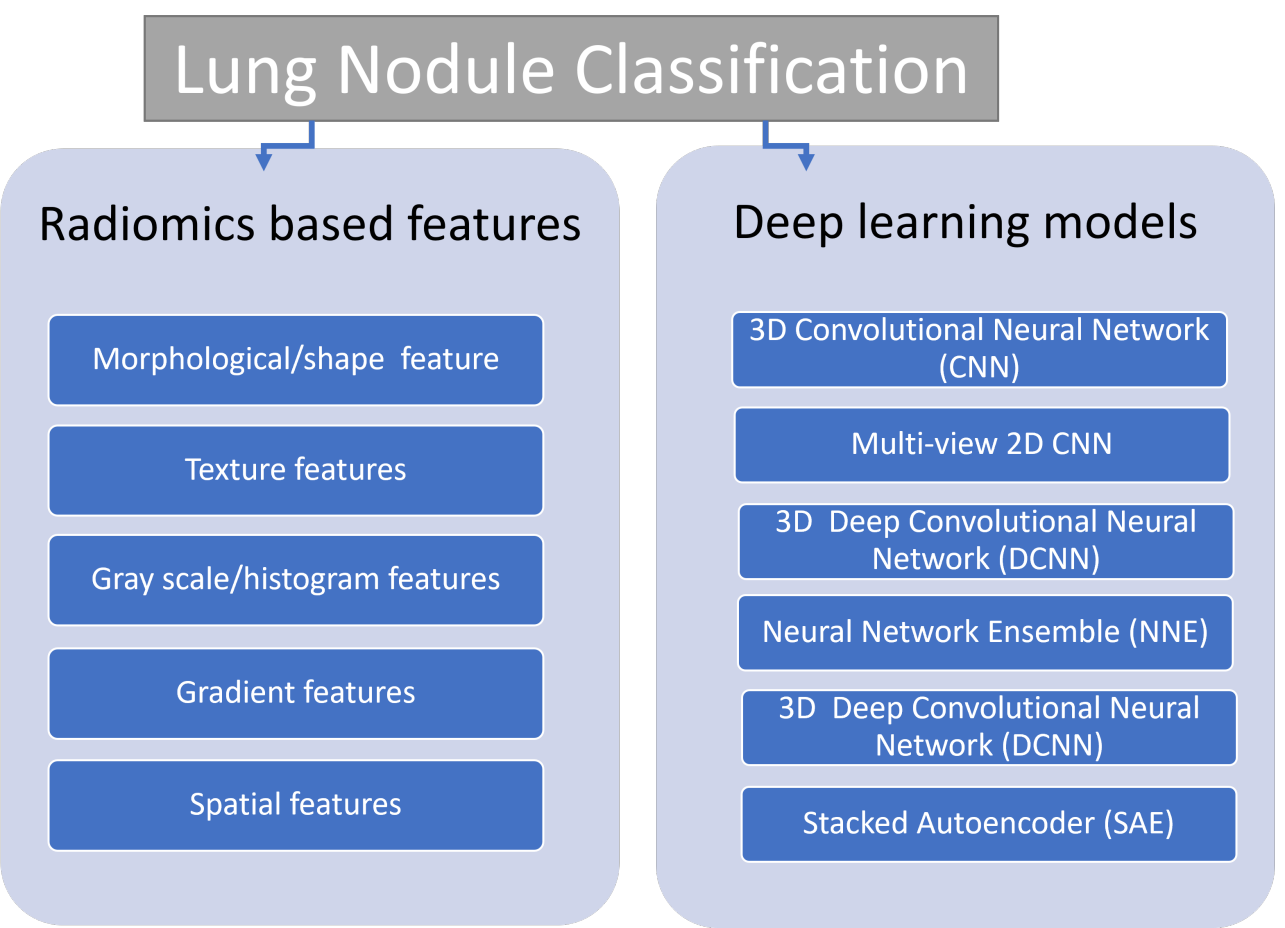
Main Categories of Lung Nodule Classification.

One of the major limitations of using CAD systems in the detection of lung nodule is the high false positive rate which hinders the accuracy and lowers its efficacy as a screening framework that could be used on a large scale population. False positive nodules are associated with extra costs and hazards as they lead to unnecessary biopsies, more prolonged follow up imaging, and extra worry by patients and their families. So, accurate classification of detected pulmonary nodule is of utmost importance to overcome these problems. After nodule detection and segmentation, comes nodule classification. TPNs are classified by two large architectures: either radiomics feature-based scheme or deep learning models [136,137,138,139] (Figure 3). The feature radiomic scheme uses different sets of features, that could be morphological/shape (spherical disproportion, circularity … etc.), texture features, gray scale/histogram features (average, standard deviation, skewness…), gradient features (average, standard deviation, kurtosis…), and spatial features (location of the nodule) [140,141]. The extracted data from image voxels are then gathered and transformed into numeric form called feature radiomics [142]. A group of numeric features (radiomics) represent what is called feature vector. Then, a classifier (which is a machine learning model) differentiates feature vectors according to training algorithms and labelled data [143]. Famous classifiers include support vector machine, and random forest [144]. The advantage of radiomics model is that it could build models of high performance out of limited datasets, yet it requires manual tumor segmentation and hand-crafted feature extraction [145,146,147].

On the other hand, classifiers are used to build end to end convolutional neural networks, fully connected neural network, or deep neural network to reach final nodule classification through semantic feature analysis [12,147,148,149,150,151]. As mentioned earlier, ML and neural networks do not require segmentation or hand-crafted feature extraction [152,153]. DNN could assess difficult cases which does not fit in the predefined feature characteristics, yet still with satisfactory results. Deep layers such as ResNet and DenseNet are usually used to train the DNN model [69,136,154,155].

The process of nodule classification requires analysis of data obtained from 3D images. However, most of the available models either use 2D data to build a 3D CNN model [156] or a multi-view 2D CNN model [157,158,159]. Uthoff et al. [156] developed a ML pipeline using k-medoids clustering and information theory to pick efficient predictor sets for different amounts of parenchyma. Their method had high sensitivity of 100% and specificity of 96%. On the other hand, Shen et al. [157] used a multiscale 2-layered CNN to diagnose lung cancer in CT chest images, reaching an accuracy of 84.86%, while Jung et al. [160] used a 3D deep convolutional neural network (DCNN) with shortcut and dense connections to classify lung nodules. These connections allow gradients to pass directly and quickly, thus overcome gradient vanishing problems. In addition to acquiring three dimensional features instead of two. Their method had higher competition performance metric (CPM) of about 0.9 as compared to other state of the art methods. Chen et al. [160] used a neural network ensemble (NNE) to evaluate lung nodules and differentiate between probably malignant, uncertain, and probably benign nodules with an accuracy of 78.7%. Another study using texture features and artificial neural networks found that feed forward back propagation showed more accurate nodule classification as compared to feed forward neural networks and that skewness was the most accurate parameter [161]. Kumar et al. [149] proposed another type of neural network for lung nodule classification called stacked autoencoder (SAE) with an accuracy of 75.01%. Wilms et al. [78] presented a model-based 4D segmentation of lungs with large tumors in 4D CT data sets in which a 4D statistical shape model is fitted to the 4D image sequence respecting inter and intra-patient variation. Ardila et al., proposed a DL model that extracts data from patient’s prior and current CT images to predict the risk of development of bronchogenic carcinoma [162]. This model had high accuracy when applied on lung cancer screening trial cases and on independent validation group. They compared their results with a group of 6 radiologists. Interestingly, their model was comparable to radiologists in the evaluation of prior and recent CT images, but it outperformed the radiologists when evaluating recent CT image only. Li et al. [163] evaluated the diagnostic performance of a CAD commercial software program called InferRead CT Lung Research (ICLR) which was based on 3D CNN. They found that ICLR had high accuracy in risk prediction of bronchogenic carcinoma unlike benign or metastatic lesions. One recent research [164] utilized a 2-level classification of pulmonary nodules into benign and malignant with further subdivision of malignant nodules into serious and mild malignant nodules using CNN with transfer learning, they attained high accuracy similar to other published research.

Other studies were more concerned in correlating between pulmonary nodules morphological features and finger print of genetic mutations of pathological types of lung cancer (radio-genomics). This is particularly important in the assessment of success of gene inhibiting therapy [164,165,166,167,168].

Regarding the diagnostic performance, a bunch of studies proved that deep leaning is superior to ML models, owing to self-learning capabilities of the later [78,149,161,162,163,164,165,166,167,168,169,170,171,172,173,174,175]. Song et al. [176] compared three types of neural networks; convolutional neural network, deep neural network, and stacked autoencoder (SAE). They found that CNN had the highest accuracy (84.15%), while another more recent study showed high accuracy (AUC of 0.99) using CNN based DL systematic approach called NoduleX [177]. Table 3 presents a summary of state-of-the-art pulmonary nodules classifications.

## 5. Limitations and Future Prospects

The scale of dataset used in CNN model is a crucial factor in the determination of whether it is a good model for training or not [182]. Collecting a large number annotated images could be a year-long process or even impossible owing to nature of medical imaging. To overcome this problem, large public datasets were introduced. Another solution is to artificially generate datasets that are similar to those used in the training of CNN. One example is the generative adversarial network (GAN) [133]. Another suggested solution is to implement transfer learning. Transfer model and LeNet5 were suggested to deal with conditions where large datasets are not available. Transfer-learning simply uses pre-existing data from source task to analyze data obtained from target task, which is useful in situations where target task has few datasets [183]. Recent study used CNN and LeNet5 to classify pulmonary nodules into benign or malignant with further sub-classification of various types of malignancies [184]. A limitation that comes along with data sharing and data transfer is the legal aspects of patient’s privacy. Another limitation is the lack of uniform terms between radiologists (for example when to describe a nodule as subsolid or non-solid) or between pathologists (minimally invasive carcinoma or carcinoma in situ), which in turn leads to non-uniform labelling of data which may affect the trained model. Of course, the solution for this problem will be the creation of a data-reporting system to unify medical terms like what happened in BI-RADS and LI-RADS. In the clinical practice, radiologists usually get benefit from clinical data to direct differential diagnosis and reach proper decision. However most of the available algorithms depend only on features derived from the images with little or no consideration to clinical data such as age, presence or absence of risk factors (smoking). Algorithms that combine clinical and imaging data are the solution to such limitation [185]. Finally, many algorithms and models are proposed but they lack generalizability and are used mainly in research works.

## 6. Conclusions

AI and its multiple arms including CAD, ML and DL are used to design complex algorithms to detect and further characterize pulmonary nodules in order to predict malignancy risk. Along the last decade, large number of radiomic features and artificial networks were proposed, each had its own advantages and drawbacks, till now no specific method gained popular acceptance to be applied on a general population. 

## Figures and Tables

**Table 1:** Literature reviews of lung segmentation system using Hounsfield unit (HU) threshold, deformable boundaries, shape models, region/edge-based models, or machine learning (ML) based methods.

Study	Method	# Subjects	System Evaluation
Amato et al. [16,17]	*1.* Grey scale thresholding *2.* Rolling ball algorithm.	17 CT patients.	The area under the ROC curve (AUC) of the system was 93%.
Hu et al. [13]	*1. * Grey scale thresholding. *2.* Dynamic programming. *3.* Morphological operations.	eight normal CT patients.	The average intrasubject change was 2.75%±2.29%.
Itai et al. [22]	*1.* Grey scale thresholding. *2.* Active contour model.	9 CT Patients.	Qualitative evaluation only.
Silveria et al. [23,24]	*1.* Grey scale thresholding. *2.* Geometric active contour. *3.* Level sets. *4.* Expectation-maximization (EM) algorithm.	Stack of chest CT slices.	Qualitative evaluation only.
Gao et al. [19]	*1.* Grey scale thresholding. *2.* Anisotropic diffusion. *3.* 3D region growing. *4.* Dynamic programming. *5.* Rolling ball algorithm.	eight CT scans.	The average overlap coefficient of the system was 99.46%.
Pu et al. [18]	*1.* Grey scale thresholding. *2.* Geometric border marching.	20 CT patients.	Average over-segmentation and under-segmentation ratio were 0.43% and 1.63%, respectively.
Korfiatis et al. [57]	*1.* k-means clustering *2.* Support vector machine (SVM)	22 CT patients.	The mean overlap coefficient of the system was higher than 89%.
Wang et al. [58]	*1.* Gray scale thresholding. *2.* 3D gray-level co-occurrence matrix (GLCM) [59,60].	76 CT patients.	The mean overlap coefficient of the system was 96.7%.
Van Rikxoort et al. [15]	*1.* Region growing. *2.* Grey scale thresholding. *3.* Dynamic programming. *4.* 3D hole filling. *5.* Morphological closing.	100 CT Patients.	The accuracy of the system was 77%.
Wei et al. [20]	*1.* Histogram analysis and connected-component labeling. *2.* Wavelet transform. *3.* Otsu’s algorithm.	nine CT patients.	The accuracy range of the system was 76.7−94.8%.
Ye et al. [21]	*1.* 3D fuzzy adaptive thresholding. *2.* Expectation–maximization (EM) algorithm. *3.* Antigeometric diffusion. *4.* Volumetric shape index map. *5.* Gaussian filter. *6.* Dot map. *7.* Weighted support vector machine (SVM) classification.	108 CT patients.	The average detection rate of the system was 90.2%.
Sun et al. [27]	*1.* Active shape model matching method. *2.* Rib cage detection method. *3.* Surface finding approach.	60 CT patients.	The Dice similarity coefficient (DSC) and mean absolute surface distance of the system were 97.5%±0.6% and 0.84±0.23, respectively.
Sofka et al. [29]	*1.* Shape model. *2.* Boundary detection.	260 CT patients.	The errors in segmenting left and right lung were 1.98±0.62 and 1.92±0.73, respectively.
Hua et al. [30]	Graph-based search algorithm.	19 pathological lung CT patients.	The sensitivity, specificity, and Hausdorff distance of the system were 98.6%±1.1%, 99.5%±0.3%, and 13.3±4.7, respectively.
Nakagomi et al. [61]	Min-cut graph algorithm.	97 CT patients	The sensitivity and Jaccard index of the system were 91.2%±13.3%, and 97.7%±1.1%, respectively.
Mansoor et al. [52]	*1.* Fuzzy connectedness segmentation algorithm. *2.* Texture-based random forest classification. *3.* Region-based and neighboring anatomy guided correction segmentation.	more than 400 CT patients.	The DSC, Hausdorff distance, sensitivity, and specificity of the system were 95.95%±0.34%, 19.65±12.84, 96.84%±1.63%, and 92.97%±0.68%, respectively.
Yan et al. [62]	Convolution neural network (CNN).	861 CT COVID-19 patients.	The system achieved DSC of 98.7% and 72.6%, sensitivity of 98.6% and 75.1%, and specificity of 99% and 72.6% for normal and COVID-19-infected lung, respectively.
Fan et al. [63]	*1.* COVID-19-infected lung segmentation convolution neural network (Inf-Net). *2.* Semi-supervised Inf-Net (Semi-Inf-Net).	100 CT images.	The DSC (sensitivity, specificity) of Inf-Net and Semi-Inf-Net were 68.2% (69.2%, 94.3%) and 73.9% (72.5%, 96%), respectively.
Oulefki et al. [64]	Multi-level entropy-based threshold approach.	297 CT COVID-19 patients.	The DSC, sensitivity, specificity, and precision of the system were 71.4%, 73.3%, 99.4%, and 73.9%, respectively.
Sharafeldeen et al. [65]	*1.* Linear combination of Gaussian. *2.* Expectation-maximization (EM) algorithm. *3.* Modified k-means clustering approach. *4.* 3D MGRF-based morphological constraints.	32 CT COVID-19 patients.	The Overlap coefficient, DSC, absolute lung volume difference (ALVD), and 95th-percentile bidirectional Hausdorff distance (BHD) were 91.76%±3.29%, 95.67%±1.83%, 2.93±2.39, and 4.86±5.01, respectively.
Zhao et al. [66]	*1.* Grey scale thresholding. *2.* 3D V-Net. *3.* Deformation module.	112 CT patients.	DSC, sensitivity, specificity, and mean surface distance error of the system were 97.96%, 98.4%, 99.54%, and 0.0318, respectively.
Sousa et al. [67]	Hybrid deep learning model, consisted of U-Net [68] and ResNet-34 [69] architectures.	385 CT patients, collected from five different datasets.	The mean DSC of the system was higher than 93%, and the average Hausdorff distance was less than 5.2.
Kim et al. [70]	Otsu’s algorithm.	447 CT patients.	Sensitivity, specificity, accuracy, AUC, and F1-score of the system were 96.2%, 97.5%, 97%, 96.8%, and 96.1%, respectively.

**Table 2:** Literature review of pulmonary nodule detection and segmentation systems.

Study	Method	# Subjects	System Evaluation
Brown et al. [97]	*1.* Priori model. *2.* Region growing. *3.* Mathematical morphology.	31 CT patients.	The accuracy of the system was 86%.
Oda et al. [95]	*1.* 3D filter by orientation map of gradient vectors. *2.* 3D distance transformation.	33 CT patients.	The accuracy of the system was 59%.
Chang et al. [82]	*1.* Cylinder filter. *2.* Spherical filter. *3.* Sphericity test.	eight CT patients.	The detection rate of the system was 90%.
Way et al. [78]	*1.* k-means clustering. *2.* 3D active contour model	96 CT patients.	Qualitative evaluation only.
Kuhnigk et al. [121]	Automatic morphological and partial volume analysis based method.	Low-dose data from 8 clinical metastasis patients.	Results of proposed method outperformed conventional methods both systematic and absolute errors were substantially reduced. Method could successfully account for slice thickness and variations of kernel reconstruction compared to conventional methods.
Zhou et al. [124]	*1.* Detection: boosted KNN with Euclidean distance measure between the non-parametric density estimates of two regions. *2.* Segmentation: analysis of 3-D texture likelihood map of nodule region.	10 ground Glass Opacity nodules.	All 10 nodules detected with only 1 false positive nodule.
Dehmeshki et al. [122]	Adaptive sphericity oriented contrast region growing on the fuzzy connectivity map of the object of interest.	*1.* Database 1: 608 pulmonary nodules from 343 scans, *2.* Database 2: 207 pulmonary nodules from 80 CT scans.	Visual inspection found that 84% of the segmented nodules were correct, while the other 16% nodules required other segmentation solutions.
Tao et al. [123]	A multi-level statistical learning-based approach for segmentation and detection of ground glass nodule.	Database: 1100 subvolumes (100 contains ground glass nodule) acquired from 200 subjects.	Classification accuracy: 92.28% (overall), and 89.87% (ground glass nodule).
Messay et al. [98]	*1.* Thresholding. *2.* Morphological operations. *3.* Fisher Linear Discriminant (FLD) classifier.	84 CT patients.	The sensitivity of the system was 82.66%.
Kubota et al. [126]	Region Growing.	*1.* LIDC 1: 23 nodule, *2.* LIDC 2: 82 nodule, *3.* A dataset of 820 nodules with manual diameter measurements.	*1.* LIDC 1: 0.69±0.18 average overlap, *2.* LIDC 2: 0.59±0.19 average overlap.
Liu et al. [128]	*1.* Selective enhancement filter [129]. *2.* Hidden conditional random field (HCRF) [130].	24 CT patients.	The sensitivity of the system was 89.3% with 1.2 false positive/scan.
Choi et al. [107]	*1.* Dot enhancement filter. *2.* Angular histograms of surface normals (AHSN). *3.* Iterative wall elimination method. *4.* Support vector machine (SVM) classifier.	84 CT patients.	The sensitivity of the system was 97.5% with 6.76 false positive/scan.
Alilou et al. [108]	*1.* Thresholding. *2.* Morphological opening. *3.* 3D region growing.	60 CT patients.	The sensitivity of the system was 80% with 3.9 false positive/scan.
Bai et al. [109]	*1.* Local shape analysis. *2.* Data-driven local contextual feature learning. *3.* Principal component analysis (PCA).	99 CT patients	The number of false positive were reduced by more than 80%.
Setio et al. [99]	*1.* Thresholding. *2.* Morphological operations. *3.* Vector supporting machine (VSM) classifier.	888 CT patients.	The sensitivity of the system was 94.1% and 98.3% with an average of 1 and 4 false positive/scan, respectively.
Bai et al. [109]	*1.* Local shape analysis. *2.* Data-driven local contextual feature learning. *3.* Principal component analysis (PCA).	99 CT patients	The number of false positive were reduced by more than 80%.
Setio et al. [99]	*1.* Thresholding. *2.* Morphological operations. *3.* Vector supporting machine (VSM) classifier.	888 CT patients.	The sensitivity of the system was 94.1% and 98.3% with an average of 1 and 4 false positive/scan, respectively.
Akram et al. [106]	*1.* Artificial neural network (ANN). *2.* Geometric and intensity-based features.	84 CT patients.	The accuracy and sensitivity of the system were 96.68% and 96.95%, respectively.
Golan et al. [111]	Deep convolutional neural network (CNN).	1018 CT patients	The sensitivity of the system was 78.9% with 20 false positive/scan.
Bergtholdt et al. [112]	*1.* Geometric features. *2.* Grayscale features. *3.* Location features. *4.* Support vector machine (SVM) classifier.	1018 CT patients.	The sensitivity of the system was 85.9% with 2.5 false positive/scan.
Sudipta Mukhopadhyay [127]	Thresholding approach based on internal texture (solid/part-solid and non-solid), and external attachment (juxta-plural and juxta-vascular).	891 nodules from (LIDC/IDRI).	Average segmentation accuracy: 99%±1 (for soild/part-solid), 98%±2 (for non-solid).
El-Regaily et al. [110]	*1.* Canny edge detector. *2.* Thresholding. *3.* Region growing. *4.* Rule-based classifier.	400 CT patients.	The accuracy, sensitivity, and specificity of the system were 70.53%, 77.77%, and 69.5%, respectively with an average of 4.1 false positive/scan.
Zhang et al. [113]	Deep believe network (DBN).	1018 CT patients.	The accuracy of system was 90%.
Wang et al. [100]	Semi-supervised extreme learning machines (SS-ELM)	1018 CT patients.	The accuracy of the system was 96.1%.
Zhao et al. [131]	*1.* 3D U-Net [132]. *2.* Generative adversarial network (GAN) [133].	800 CT scans.	Qualitative evaluation only.
Charbonnier et al. [125]	Subsolid nodule segmentation using voxel classification that eliminated blood vessels.	170 subsolid nodules from the Multicentric Italian Lung Disease trial.	92.4% of segmented vessels, and 80.6% of segmented solid core were accepted observers.
Luo et al. [134]	3D sphere center-points matching detection network (SCPM-Net).	888 CT scans.	The sensitivity of the system was 89.2%.
Yin et al. [135]	Squeeze and attention, and dense atrous spatial pyramid pooling U-Net (SD-U-Net).	2236 CT slices.	The Dice similarity coefficient (DSC), sensitivity, specificity, and accuracy of the system were 86.96%, 89.88%, 99.32%, and 99.06%, respectively.
Bianconi et al. [120]	*1.* 12 conventional semi-automated methods (Active contours (MorphACWE, MprphGAC), cluserting (K-means, SlIC), graph-based (Felzenszwalb), region-growing (flood fill), thresholding (Kapur, Kittler, Otsu, MultiOtsu, others (MSER, Watershed)), and *2.* 12 deep learning semi-automated methods (12 CNNS designed using 4 standard segmentation models (FPN, LinkNet, PSPNet, U-Net) and 3 well-known encoders (InceptionV3, MobileNet, ResNet34)).	*1.* Dataset 1: 383 images from a cohort of 111 patients. *2.* Dataset 2: 259 images from a cohort of 100.	Semi-automated deep learning methods outperformed the conventional methods. DSCs of the deep learning based methods recorded 0.853 and 0.763 for dataset 1, and dataset 2 respectively. Conventional methods recorded DSCs of 0.761 and 0.704.

**Table 3:** Literature review of pulmonary nodule classification systems.

Study	Method	# Subjects	System Evaluation
Dehmeshki et al. [148]	Shape-based region growing.	3D lung CT data where nodules are attached to blood vessels or lung wall.	Qualitative evaluation only.
Lee et al. [169]	Commercial CAD system (IQQA-Chest, EDDA Technology, Princeton Junction, NJ, USA).	200 chest radiographs (100 normal, 100 with malignant solitary nodules.	Sensitivity of 87%, false positive rate of 0.19.
Kuruvilla et al. [161]	Feed forward and feed forward back propagation neural networks.	155 patients from LIDC	Classification accuracy of 93.3%.
Yamamoto et al. [165]	Random forest.	172 patients with NSCLC.	Sensitivity of 83.3%, specificity of 77.9%, accuracy of 78.8% in independent testing.
Orozco et al. [147]	*1.* Wavelet feature descriptor, 2. SVM.	45 CT scans from ELCAP and LIDC.	Total preciseness in classifying cancerous from non-cancerous nodules was 82%; sensitivity of 90.90%, and specificity of 73.91%.
Kumar et al. [149]	Deep Features using autoencoder.	4323 nodules from NCI-LIDC dataset.	75.01% overall accuracy, 83.35% sensitivity, and false positive of 0.39/patient (10-fold cross validation).
Hua et al. [175]	*1.* A deep belief network (DBN), *2.* CNN.	LIDC	Sensitivity (DBN: 73.4%, CNN: 73.3%), Specificity (DBN: 82.2%, CNN: 78.7%).
Kang et al. [171]	3D multi-view CNN (MV-CNN).	LIDC-IDRI	Error rate of 4.59% for binary classification (benign and malignant) and 7.70% for ternary classification(benign, primary malignant and metastatic malignant).
Ciompi et al. [173]	Multi-stream multi-scale convolutional networks.	*1.* Italian MILD screening trial, *2.* Danish DLCST screening trial.	Best accuracy of 79.5%.
Song et al. [176]	*1.* CNN, *2.* Deep neural network (DNN), *3.* Stacked autoencoder (SAE).	LIDC-IDRI	Accuracy of 84.15%, sensitivity of 83.96%, and specificity of 84.32%.
Tajbakhsh et al. [138]	*1.* Massive training artificial neural networks (MTANN), *2.* CNN.	LDCT acquired from 31 patients.	AUC = 0.8806 (95% confidence interval (CI): 0.8389–0.9223).
Li et al. [145]	Support vector machine (SVM).	248 GGNs.	Accuracy of classifying GGNs into atypical adenomatous hyperplasia (AAH), adenocarcinoma in situ (AIS), minimally invasive adenocarcinoma (MIA), and invasive adenocarcinoma (IA) was 70.9%. Accuracy of classification between AIS and MIA nodules is 73.1%, and between indolent versus invasive lesions is 88.1%.
Huang et al. [154]	Dense convolutional network (DenseNet).	*1.* CIFAR, *2.* SVHN, *3.* ImageNet.	Error rates for CIFAR (C10: 5.19%, C10+: 3.46%, C100: 19.64%, C100+: 17.18%), SVHN (1.59%), ImageNet (error rates with single-crop (10-crop) are: top-1 (25.02 (23.61), 23.80 (22.08), 22.58 (21.46), 22.33 (20.85)), top-5 (7.71 (6.66), 6.85 (5.92), 6.34 (5.54), 6.15 (5.30))).
Nibali et al. [158]	ResNet	LIDC/IDRI	Sensitivity of 91.07%, specificity of 88.64%, precision of 89.35%, AUC of 0.9459, and accuracy of 89.90%.
Liu et al. [159]	Multi-view multi-scale CNNs	LIDC-IDRI and ELCAP	Classification rate as 92.1%.
Zhao et al. [152]	A deep learning system based on 3D CNNs and multitask learning	651 nodules with labels of AAH, AIS, MIA, IA.	Classification accuracy using 3 class weighted average F1 score is: 63.3% compared to radiologists who achieved 55.6%, 56.6%, 54.3%, and 51.0%.
Li et al. [150]	Multivariable linear predictor model built on semantic features.	100 patients from NLST-LDCT.	AUC at baseline screening: 0.74, at first followup: 0.88, and at second followup: 0.96.
Lyu et al. [172]	Multi-level CNN (ML-CNN).	LIDC, IDRI (1018 cases from 1010 patients)	Accuracy: 84.81%.
Shaffie et al. [174]	*1.* Seventh-order Markov Gibbs random field (MGRF) model [178,179,180], *2.* Geometric features, *3.* Deep autoencoder classifier.	727 nodules from 467 patients (LIDC).	Classification accuracy of 91.20%.
Causey et al. [177]	Deep learning CNN.	LIDC-IDRI	Accuracy of malignancy classification with AUC of approximately of 0.99.
Uthoff et al. [156]	k-medoids clustering and information theory.	Training: (74 malignant, 289 benign), Validation (50 malignant, 50 benign).	AUC = 0.965, 100% sensitivity and 96% specificity.
Ardila et al. [162]	A deep learning CNN.	6716 National Lung Cancer Screening Trial cases, independent clinical validation set of 1139 cases.	AUC = 94.4%.
Liu et al. [151]	*1.* Multivariate logistic regression analysis, *2.* Least absolute shrinkage and selection operator (LASSO).	Benign and malignant nodules from 875 patients.	Training: AUC = 0.836; 95% CI: 0.793–0.879) and validation (AUC = 0.809; 95% CI: 0.745–0.872).
Gong et al. [136]	A deep learning–based artificial intelligence system for classifying ground-glass nodule(GGN) into invasive adenocarcinoma (IA) or non-invasive IA.	828 GGNs of 644 patients (209 are IA and 619 non-IA, including 409 adenocarcinomas in situ and 210 minimally invasive adenocarcinomas).	AUC = 0.92±0.03.
Sim et al. [137]	Radiologists assisted by deep learning–based CNN.	600 lung cancer–containing chest radiographs and 200 normal chest radiographs.	Average sensitivity improved from 65.1% to 70.3%, and number of false positives per radiograph declined from 0.2 to 0.18.
Wang et al. [153]	A two-stage deep learning strategy: prior-feature learning followed by adaptive-boost deep learning.	1357 nodules (765 noninvasive (AAH and AIS) and 592 invasive nodules (MIA and IA)).	Classification accuracy of 73.4%±1.4 compared to specialists who achieved 69.1%, 69.3%, and 67.9%. AUC= 81.3%±2.2.
Xia et al. [155]	1. Recurrent residual CNN based on U-Net, 2. Information fusion method.	373 GGNs from 323 patients.	AUC= 0.90±0.03, accuracy: 80.3%.
Li et al. [163]	CLR software based on 3D CNN with DenseNet architecture as a backbone.	486 consecutive resected lung lesions(320 adenocarcinomas, 40 other malignancies, 55 metastases, and 71 benign lesions).	Classification accuracy for adenocarcinomas, other malignancies, metastases, and benign lesions was 93.4%, 95.0%, 50.9%, and 40.8%, respectively.
Hu et al. [139]	*1.* 3D U-NET, *2.* Deep neural network.	513 GGNs (100 benign, 413 malignant).	Accuracy of 75.6%, F1 score of 84.6%, weighted average F1 score of 70.3%, and Matthews correlation coefficient of 43.6%.
Farahat et al. [181]	1. Three MGRF energies, extracted from three different grades of COVID-19 patients, 2. Artificial neural network.	76 CT COVID-19 patients.	100% accuracy, and 100% Cohen kappa.

## Data Availability

Not applicable.

## Abbreviations

The following abbreviations are used in this manuscript:

HU	Hounsfield Unit
ABM	Adaptive Border Marching
A-CNN	Amalgamated Convolutional Neural Network
ASM	Active Shape Model
CAD	Computer-Aided Diagnosis
CADe	Computer-Aided Detection System
CADx	Computer-Aided Diagnosis System
DL	Deep Learning
CNN	Convolutional Neural Network
MV-CNN	Multi-view CNN
ML-CNN	Multi-level CNN
AHSN	Angular Histograms of Surface Normals
CPM	Competition Performance Metrics
CT	Computed Tomography
CV	Chan Vese
DBN	Deep Belief Network
DCNN	Deep Convolutional Neural Network
DNN	Deep Neural Network
ELM	Extreme Learning Machines
FLD	Fisher Linear Discriminant
FPN	False Positive Nodule
GAN	Generative Adversarial Network
GGO	Ground Glass Opacity
GGN	Ground Glass Nodule
ICLR	InferRead CT Lung Research
KB	Knowledge Bank
k-NN	K-nearest Neighbor
LDA	Linear Discriminate Analysis
LDCT	Low Dose Computed Tomography
LIDC-IDRI	Lung Image Database Consortium and Image Database Resource Initiative
MGRF	Markov Gibbs Random Field
ML	Machine Learning
MPP	Multi Player Perception
NNE	Neural Network Ensemble
PNN	Probabilistic Neural Network
RASM	Robust Active Shape Model
ROI	Region of Interest
RPCA	Robust Principal Component Analysis
SAE	Stacked Autoencoder
SS-ELM	Semi-Supervised Extreme Learning Machines
SVM	Support Vector Machine
TPN	True Positive Nodule
AUC	Area Under the Curve
IA	Invasive Adenocarcinoma
MTANN	Massive training artificial neural networks
NCI	National Cancer Institute
SVHN	Street View House Numbers Dataset
LASSO	Least Absolute Shrinkage and Selection Operator
AAH	Atyoical Adenomatous Hyperplasia
MIA	minimally invasive adenocarcinoma
AIS	Adenocarcinoma in Situ
GLCM	Gray-Level Co-occurrence Matrix
EM	Expectation–maximization method
DSC	Dice Similarity Coefficient
Inf-Net	COVID-19-infected lung segmentation convolution neural network
Semi-Inf-Net	semi-supervised Inf-Net
ALVD	absolute lung volume difference
BHD	bidirectional Hausdorff distance
HCRF	Hidden conditional random field
SCPM-Net	sphere center-points matching detection network
SD-U-Net	Squeeze and attention, and dense atrous spatial pyramid pooling U-Net
